# Fructose Metabolism in Cancer

**DOI:** 10.3390/cells9122635

**Published:** 2020-12-08

**Authors:** Nils Krause, Andre Wegner

**Affiliations:** Department of Bioinformatics and Biochemistry, BRICS, Technische Universität Braunschweig, 38106 Braunschweig, Germany; nils.krause@tu-braunschweig.de

**Keywords:** fructose metabolism, cancer metabolism, polyol pathway, pentose phosphate pathway, lipogenesis, AKR1B1, KHK, SORD, HFCS

## Abstract

The interest in fructose metabolism is based on the observation that an increased dietary fructose consumption leads to an increased risk of obesity and metabolic syndrome. In particular, obesity is a known risk factor to develop many types of cancer and there is clinical and experimental evidence that an increased fructose intake promotes cancer growth. The precise mechanism, however, in which fructose induces tumor growth is still not fully understood. In this article, we present an overview of the metabolic pathways that utilize fructose and how fructose metabolism can sustain cancer cell proliferation. Although the degradation of fructose shares many of the enzymes and metabolic intermediates with glucose metabolism through glycolysis, glucose and fructose are metabolized differently. We describe the different metabolic fates of fructose carbons and how they are connected to lipogenesis and nucleotide synthesis. In addition, we discuss how the endogenous production of fructose from glucose via the polyol pathway can be beneficial for cancer cells.

## 1. Introduction

Fructose occurs naturally in many fruits and vegetables, where it is often bonded to glucose to form the disaccharide sucrose. Hence, it is an integral part of the human diet. With the discovery of the enzymatic conversion of glucose to fructose in 1957 [1], fructose is used as a supplement in the food industry. Fructose is much sweeter than other hexoses, and, therefore, is widely used as a sweetener in processed food and beverages. Since 1970, fructose has been used in the form of high fructose corn syrup (HFCS) leading to an increase of fructose intake in the human diet in the last decades [2]. Fructose consumption, as the combined intake of sucrose and HFCS, increased from 64 g/day in 1970 to 81 g/day in 1997 in the US. The intake of fructose as monosaccharide increased from less than 0.5 g/day to more than 40 g/day [3]. In the US, the sole HFCS intake increased from 0.4 g/day *per capita* in 1970 to a maximum of 46.6 g/day in 1998, and reduced to 26.8 g/day of HFCS in 2019 [4].

In order to understand the role of fructose in cancer cell metabolism, it is necessary to know and understand how healthy cells metabolize fructose (e.g., the involved metabolic pathways). In the following, we will describe the metabolic fates of fructose in humans and the key enzymes involved in fructose metabolism. Furthermore, we give a short overview of known correlations between fructose consumption and non-cancer diseases like obesity, diabetes, and metabolic syndrome, as they are major risk factors for the development of many different cancer types [5,6], also reviewed in [7]. Subsequently, we will highlight the link between fructose metabolism and cancer cell proliferation, focusing on how fructose is metabolized in different types of cancer, which enzymes are involved, and how fructose metabolism is associated with cancer development and outcome.

## 2. Physiological Fate(s) of Fructose

The degradation of fructose is usually referred to as fructolysis and is defined as the metabolism of fructose from dietary sources. Although fructolysis shares many of the enzymes and metabolic intermediates with glucose metabolism through glycolysis, glucose and fructose are metabolized differently. While glucose is directly metabolized by virtually every cell type in the human body, fructolysis is assumed to be mainly restricted to the liver. However, small oral loads are metabolized in the small bowel and in small parts extracted by the kidney and other tissues [8]. In the liver, fructose is transported across membranes via passive transport. Here, glucose transporter 2 (GLUT2) is the main fructose transporter. Besides fructose, GLUT2 can also transport glucose and galactose [9]. The sole known fructose specific transporter in the family of glucose transporters is GLUT5, encoded by the gene SLC2A5 and localized mainly in the small intestine [10,11]. While several GLUT transporters are able to transport fructose, GLUT5 is specific for fructose with no ability to transport glucose or galactose [12,13].

**Figure 1:**
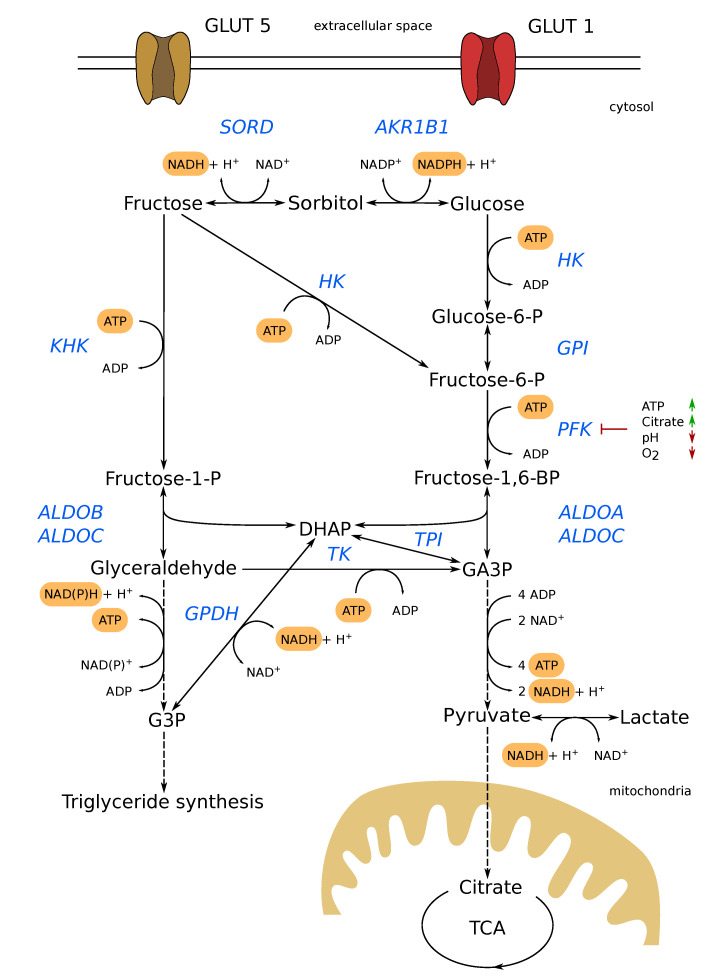
Overview of fructose and glucose degradation pathways. Fructose uptake is mediated by GLUT5 and can be metabolized via different pathways: (1) phosphorylation via KHK to F1P, which is then hydrolyzed by aldolase B to DHAP and GA. Subsequently, fructose carbons can therefore enter glycolysis and the pentose phosphate pathway. (2) Phosphorylation via HK, entering glycolysis in the form of F6P, and (3) via the polyol pathway producing glucose which in turn can enter gylcolysis. Glucose can be taken up by several glucose transporters (here GLUT1) and is metabolized via glycolysis. Glucose can also be used for endogenous fructose production providing the possibility to bypass PFK. PFK is the rate-limiting enzyme of glycolysis and is inhibited by high levels of ATP and citrate as well as low pH and low oxygen levels. From an energetic point of view, the degradation pathways from fructose and glucose to pyruvate do not differ. Fructose degradation via KHK to GA3P consumes two ATP just as glucose in glycolysis. In the pay off phase, both pathways generate four ATP and two NADH+H^+^. Bypassing PFK inhibition by endogenous fructose production, however, consumes one additional NADPH+H^+^ and produces one NADH+H^+^. AKR1B1: aldo-keto reductase family 1, member B1; ALDO: aldolase; G3P: glycerol 3-phosphate; GA3P: glyceraldehyde 3-phosphate; GLUT1: glucose transporter 1; GLUT5: fructose transporter; GPDH: glycerol 3-phosphate dehydrogenase; GPI: glucose-6-phosphate isomerase; HK: hexokinase; KHK: keto hexokinase; PFK: phosphofructokinase; SORD: sorbitol dehydrogenase; TCA: tricarboxylic acid cycle; TK: triose kinase; TPI: triose-phosphate isomerase.

The ingested fructose is mainly metabolized via ketohexokinase (KHK), also known as fructokinase in the liver. KHK phosphorylates fructose to fructose-1-phosphate (F1P), which is then hydrolyzed via aldolase B to dihydroxyacetone phosphate (DHAP) and glyceraldehyde (GA) (Figure 1). Both DHAP and GA can be further metabolized via glycolysis in the form of glyeraldehyde-3-phosphate (GA3P). Furthermore, GA can be metabolized to glycerol, which in turn can be phosphorylated to glycerol-3-phosphate (G3P), which can be utilized for lipid synthesis or transporting reducing equivalents into mitochondria via the glycerol-3-phosphate shuttle [14]. Beyond that, fructose can be phosphorylated via hexokinase (HK) to fructose-6-phosphate (F6P). Entering glycolysis, F6P is phosphorylated to fructose-1,6-bisphosphate (F1,6BP) via phosphofructokinase (PFK). Like F1P, F1,6BP is hydrolyzed via aldolase, but this time in DHAP and GA3P (Figure 1). The advantage of fructose degradation via KHK is to bypass PFK. PFK is the rate-limiting enzyme of glycolysis and is inhibited by high levels of ATP and citrate as well as low pH and low oxygen levels. Besides these two pathways with an initial phosphorylation step, fructose can theoretically also be converted into glucose. The so-called polyol pathway consists of two reversible reactions, with sorbitol as an intermediate (Figure 1). Here, the hydrogenation of fructose to sorbitol is catalyzed by sorbitol dehydrogenase (SORD) oxidizing NADH to NAD^+^. Subsequently, sorbitol is dehydrogenated to glucose via aldo-keto reductase family 1, member B1 (AKR1B1). This reaction needs NADP^+^ as a cofactor. Since the direction of a metabolic reaction depends on the metabolic levels of its substrates and products [15], the polyol pathway provides the means to produce fructose from glucose endogenously [16], as it is known in diabetic patients.

In summary, for the degradation of fructose, three enzymes seem to be essential: KHK phosphorylating fructose to F1P, HK phosphorylating fructose to F6P, and SORD hydrogenating fructose to sorbitol.

## 3. Fructose in Disease

Although we will focus on the link between fructose metabolism and cancer, we will briefly mention other diseases affected by fructose metabolism, as some of them are known risk factors for cancer development. For more details, we refer to other reviews [7,17,18,19]. To date, there are three known inborn errors in fructose metabolism: essential or benign fructosuria due to KHK deficiency; hereditary fructose intolerance and fructose-1,6-bisphosphatase (FBPase) deficiency [19].

Essential fructosuria is caused by a loss of function mutation in KHK. This genetic disorder is asymptotic and harmless. Here, ingested fructose is partly excreted in the urine, while the rest is metabolized via hexokinase to F6P in extrahepatic tissue, like adipose tissue and muscle [19,20].

Hereditary fructose intolerance is caused by a deficiency of aldolase B, which converts F1P into DHAP and GA. Loss of aldolase B in combination with a high KHK activity leads to an accumulation of F1P. The high KHK activity depletes the intracellular ATP levels. This lack of ATP leads to increased uric acid production and a release of magnesium, which consequently are a cause for hepatic and renal dysfunction. These toxic effects in hereditary fructose intolerance can be fatal, when sorbitol and fructose are not removed from the diet [19,21,22].

FBPase deficiency is a rare, autosomal recessive disorder affecting one of the key enzymes of gluconeogenesis. Like hereditary fructose intolerance, FBPase deficiency results in F1P accumulation, with severe neurological complications induced by ingesting fructose or glycerol [19,20,23]. Due to the FBPase deficiency, its substrate F1,6BP accumulates, which in turn leads to higher levels of trioses and product inhibition of the aldolase reaction [12,13,20].

In addition to the genetic defects in fructose metabolism, there are several metabolic diseases associated with increased fructose intake. Over the last few years, there were several studies describing the link between fructose consumption and the development of obesity [3,24,25,26,27]. Increased fructose intake leads to an increased energy intake, which in combination with an increased lipid synthesis results in obesity. Beyond that, fructose plays an important role in diabetes and metabolic syndrome, as an increased fructose consumption can lead to insulin resistance [28,29,30].

## 4. Fructose in Cancer Metabolism

The link between cancer and altered glucose metabolism was already discovered by Otto Warburg around 100 years ago. While aberrant glucose metabolism and the Warburg effect are well investigated [31], the connection between cancer and fructose metabolism needs to be investigated in more detail [7]. Fructose can be metabolized in many different ways (Figure 1). Some of these metabolic pathways overlap with those of glucose degradation and play a central role in cell growth and survival. Therefore, it is not surprising that fructose, like glucose, can affect the growth, proliferation, and survival of cancer cells [32,33]. Before we discuss important metabolic pathways of fructose metabolism in more detail, we will shortly highlight recent studies connecting the fructose transporter GLUT5 to cancer development and progression. In patients with lung adenocarcinoma, a poor prognosis is correlated with an upregulation of GLUT5 [34]. The authors demonstrate that the depletion of GLUT5 attenuated cell proliferation and invasion, and increased apoptosis. The overexpression of GLUT5, on the other hand, enhanced cell proliferation, migration, invasion, and tumorigenesis [34]. Enhanced fructose utilization mediated by GLUT5 was also found in acute myeloid leukemia. The increased fructose uptake resulted in an increased proliferation rate, colony growth, and enhanced migration and invasion. Consequently, high expression of GLUT5 and enhanced fructose utilization are associated with poor outcomes and exacerbate leukemic phenotypes [11]. In addition, GLUT5 is overexpressed in several types of cancer, e.g., glioblastoma, colon, liver, lung, breast, and prostate [35,36].

### 4.1. Polyol Pathway

The polyol pathway describes the two-step conversion of glucose to fructose [37,38,39]. First, glucose is reduced to sorbitol with the use of NADPH, then sorbitol is oxidized to fructose producing NADH from NAD^+^. The polyol pathway is well known for its pathological implications in diabetes [40,41,42,43]. Here, in hyperglycemic state, sorbitol sythesis increases, thereby consuming most of the NADPH, which in turn results in increased oxidative stress [40,43,44,45]. The polyol pathway is interesting for fructose metabolism because it provides the means to either convert fructose to glucose or to endogenously produce fructose from glucose. Both of these options provide an interesting perspective on cancer cell metabolism.

Converting fructose to glucose via the polyol pathway can theoretically directly fuel glycolysis. This would enable fructolysis detached from KHK. In humans, the conversion of fructose via KHK is assumed to be mainly restricted to liver [46,47]. However, in tumors, KHK is apparently expressed more frequently [48,49,50]. To our knowledge, there is no experimental evidence that this pathway is physiologically relevant. Converting fructose to glucose via the polyol pathway would require low glucose and low NADPH levels. The reverse flux, from glucose to fructose, enabling endogenous fructose production, seems to be more physiologically relevant [51,52]. It is important to note that the endogenous fructose production with subsequent KHK activity bypasses the rate-limiting step in glycolysis, catalyzed by PFK, en route to GA3P production. The phosphorylation of F6P to F1,6BP mediated by PFK is inhibited by high levels of ATP or citrate and a low pH (Figure 1). This bypassing effect was recently observed in naked mole-rats by Park et al. [53]. In their study, the authors observed that naked mole-rats can survive a prolonged time under low oxygen levels and even anoxia because they are able to utilize fructose to fuel glycolysis for ATP generation. Low oxygen levels usually lead to an inhibition of oxidative phosphorylation and an increase in glycolytic activity and subsequently to a lower pH, which in turn would inhibit PFK. The authors concluded that fueling glycolysis with fructose via KHK avoided the PFK feedback inhibition by low pH, as GA and DHAP enter glycolysis downstream of PFK (Figure 1). Expanding on that concept, it is not too far fetched that cancer cells would employ such a mechanism. Most solid tumors have an inadequate supply of oxygen, resulting in hypoxic and an acidic environment [54]. In that context, polyol pathway activity could provide the means for cancer cells to produce fructose from glucose, bypassing PFK and increasing the glycolytic rate.

A direct link between polyol pathway activity and cancer was discovered by Schwab et. al [55]. In their study, the authors showed a strong correlation between AKR1B1 expression and epithelial-to-mesenchymal transition (EMT) in lung cancer patients and in an EMT-driven colon cancer mouse model. In in vitro experiments, they detected increased AKR1B1 levels in mesenchymal-like cancer cells, while AKR1B1 knockdown was sufficient to revert EMT. They observed similar results in EMT suppression when they targeted SORD. Furthermore, Schwab and colleagues showed that glucose-induced activation of the polyol pathway controls EMT via TGFβ autocrine stimulation. TGFβ levels in AKR1B1 and SORD knockdown cells were reduced and EMT markers could be rescued adding exogenous TGFβ [55]. In line with these results, there are a couple more studies pointing towards a correlation between the polyol pathway, in particular AKR1B1, and cancer. AKR1B1 is overexpressed in breast, ovarian, cervical, and rectal cancer [56]. In colorectal cancer cells, AKR1B1 affects cellular proliferation and cell cycle progression. In addition, AKR1B1 enhances cell motility and NF-κB activity, resulting in a poor prognosis in colorectal cancer patients [57].

While there is mounting experimental evidence showing the connection of aldo reductases and cancer, little is known about the connection between SORD and cancer. In addition to the findings by Schwab and coworkers showing the association between SORD expression and EMT [55], Uzozie and colleagues analyzed SORD overexpression in precancerous colorectal neoplasms [58]. Here, increased SORD activity in adenomas and cancer cell lines lead to changes in expression of AKR1B1 and KHK. When SORD is upregulated, AKR1B1 was decreased and KHK was upregulated.

In summary, endogenous fructose production via the polyol pathway seems to be an important part of cancer cell metabolism. The underlying molecular mechanism, however, remains elusive. Besides bypassing PFK regulation and entering glycolysis for ATP production, endogenously produced fructose could enter several other pathways important for cancer cell physiology such as the hexosamine pathway [59], the pentose phosphate pathway [60], or de novo lipogenesis [61].

### 4.2. The Pentose Phosphate Pathway

**Figure 2:**
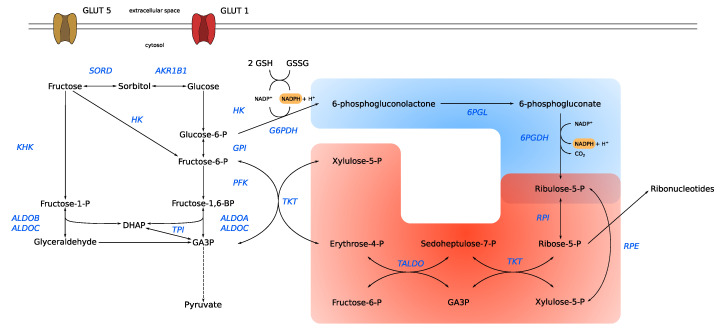
Fructose contribution to the pentose phosphate pathway. Fructose can contribute to the oxidative (blue) as well as the non-oxidative (red) pentose phosphate pathway (PPP). To enter the oxidative PPP, fructose has to be either metabolized via the polyol pathway to glucose and further to G6P or directly via HK to F6P which can be converted to G6P. Direct phosphorylation via HK to F6P provides also a substrate for the non-oxidative PPP. Together with GA3P, F6P is converted by TKT to X5P and E4P. GA3P can also be provided by fructose metabolized via KHK. Both F6P and GA3P are used in further reactions of the non-oxidative PPP, demonstrating multiple entry points of fructose into the non-oxidative PPP. AKR1B1: aldo-keto reductase family 1, member B1; ALDO: aldolase; E4P: erythrose 4-phosphate; F6P: fructose 6-phosphate; G3P: glycerol 3-phosphate; GA3P: glyceraldehyde 3-phosphate; GLUT1: glucose transporter 1; GLUT5: fructose transporter; GPI: glucose-6-phosphate isomerase; HK: hexokinase; KHK: keto hexokinase; PFK: phosphofructokinase; SORD: sorbitol dehydrogenase; TCA: tricarboxylic acid cycle; TPI: triose-phosphate isomerase. TKT: transketolase; TALDO: transaldolase; G6PDH: glucose-6-phosphate deyhdrogenase; GSH: glutathione; GSSG: glutathione disulfide; 6PGL: 6-phosphogluconolactonase; 6PGDH: 6-phosphogluconate dehydrogenase; RPE: ribulose 5-phosphate 3-epimerase; RPI: ribose-5-phosphate isomerase; X5P: xylulose 5-phosphate.

In rapidly dividing cells, such as cancer cells, it has been shown that the pentose phosphate pathway (PPP) is critical to sustain high proliferation rates [62] and that fructose metabolism plays an important role in it [63]. Generally, the PPP has two important cellular functions: (1) to provide metabolic precursors for macromolecule synthesis (ribose 5-phosphate for nucleotide and erythrose 4-phosphate for amino acid biosynthesis) and (2) to provide NADPH for anabolism and redox homeostasis. It can be broken down into two branches: the oxidative and the non-oxidative branch (Figure 2). While the oxidative branch has three irreversible reactions, the non-oxidative branch comprises a series of reversible reactions that connect the PPP to glycolysis.

In cancer cells, most of the pentose phosphates, used for de novo nucleotide synthesis, are derived from the PPP [62]. As described above (Figure 1), fructose can be metabolized in various ways, and, therefore, can potentially enter the oxidative as well as the non-oxidative branch of the PPP. However, it has been shown that fructose is preferentially metabolized via the non-oxidative branch in pancreatic cancer cells [63]. As such, the fructose carbon backbone can enter the non-oxidative branch as glyceraldehyd-3-phosphate or fructose-6-phosphate, catalyzed by the transketolase (TKT) enzyme (Figure 2). The authors showed that, in fructose treated cells, TKT protein expression increased 200% compared to glucose treated cells. In line with that observation, the authors observed that ${}^{13}$C labeled fructose was metabolized at a 250% higher rate than glucose via TKT to synthesize nucleic acids [63]. Using the non-oxidative branch of the PPP for ribose synthesis has the advantage that it is uncoupled from the generation of NADPH. This is useful when the metabolic requirement of ribose synthesis exceeds that of NADPH. Moreover, the non-oxidative branch has a neutral carbon balance as it is not producing CO_2_ like the oxidative branch (Figure 2).

The upregulation of TKT, and thus acceleration of the non-oxidative PPP, seems to be a general feature of cancer cells [64,65], even with glucose as the carbon source. Based on ${}^{13}$C-tracer experiments, it was shown in vitro that around 80% of ribonucleotides are derived from the non-oxidative PPP [66]. In chronic myeloid leukemia, it was shown that a HIF-1α dependent induction of TKT is associated with imatinib-resistance [67]. Inhibition of TKT activity with oxythiamine enhanced imatinib sensitivity of tumor cells [67]. In addition, a homologue of TKL, TKL-like protein (TKLL1) was detected in tumor tissue and its expression level has been correlated with cancer progression [68,69]. Although it was shown that TKLL1 possesses TKT activity, it is debated whether TKLL1 participates in the PPP [70].

It is worth underlining the importance of the non-oxidative branch for cancer cells, and it was shown that a G6PD deficiency does not reduce the risk of getting cancer [71], showing that NADPH requirements can be fulfilled by other pathways (e.g., serine-driven one-carbon metabolism [72]) and that the non-oxidative branch is sufficient to sustain nucleotide synthesis. As stated above, it is assumed that most of the ribonucleotides are derived from the non-oxidative branch and that fructose is preferentially metabolized via the non-oxidative branch of the PPP. This raises the possibility that cancer cells use glucose for endogenous fructose production via the polyol pathway and then subsequently use the fructose to fuel the non-oxidative PPP for nucleic acid synthesis.

### 4.3. De Novo Lipogenesis

Besides an increased demand of ribonucleotides, proliferating cancer cells require a constant supply of fatty acids for the biogenesis of more complex lipids (e.g., membrane lipids) [73]. Cancer cells can either increase their uptake of lipids or increase the metabolic flux into de novo lipogenesis (DNL) [74]. Generally, DNL refers to the metabolic pathway that synthesizes fatty acids from excess carbohydrates, which are then subsequently incorporated into triglycerides for energy storage and later oxidation. In humans, DNL mainly takes place in liver and adipose tissue and is mediated by the cytosolic enzymes acetyl-CoA carboxylase (ACC) and fatty acid synthase (FASN) [61]. In many cancer types, FASN is overexpressed [75], and thus the majority of fatty acids in these cells are assumed to be derived from DNL [76,77,78].

**Figure 3:**
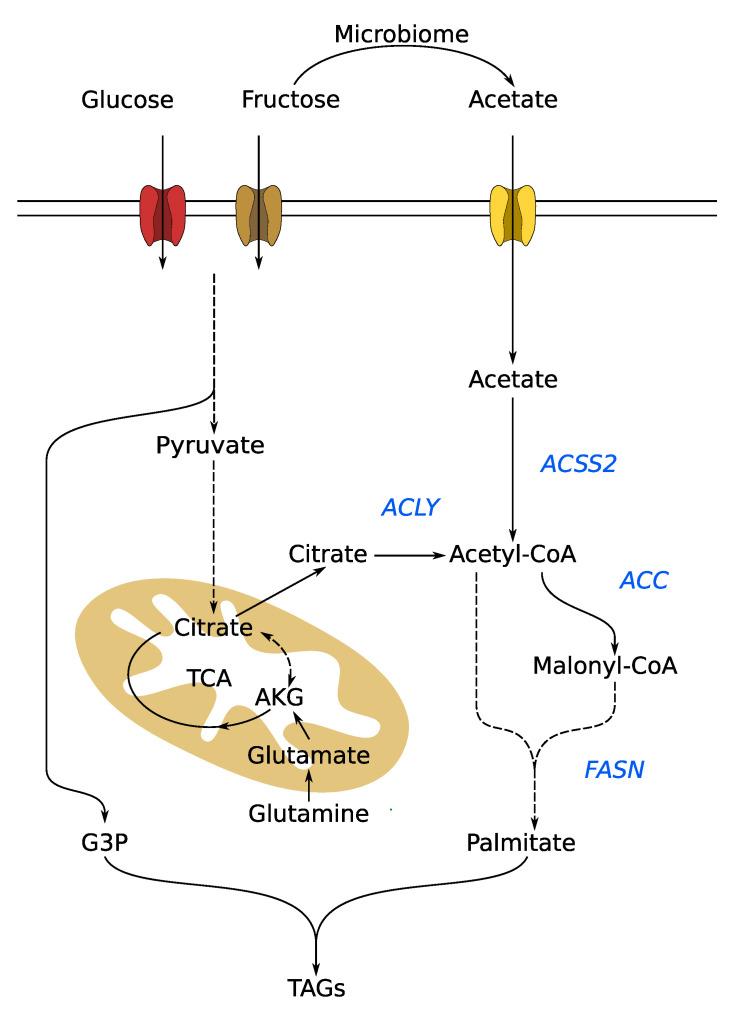
De novo lipogenesis driven by fructose and glucose carbon. Both fructose and glucose can provide carbon for DNL. On the one hand, they can end up in citrate, which is then cleaved by ACLY to provide acetyl-CoA for fatty acid synthesis. On the other hand, they can provide G3P for triacylgyceride synthesis. Besides citrate, cytosolic acetyl-CoA can also be derived from acetate. It was shown that the microbiome of mice converts fructose to acetate. This microbiota-derived acetate is then taken up and utilized for DNL in mice [81]. ACC: acetyl-CoA carboxylase; ACSS2: acyl-coenzyme A synthetase short-chain family member 2; ACLY: ATP citrate lyase; AKG: α-ketoglutarate; FASN: fatty acid synthase; G3P: glycerol 3-phosphate; TCA: tricarboxylic acid cycle; TAG: triacylglyceride.

Fatty acid synthesis starts with the initialization of a new acyl chain by FASN from acetyl-CoA which is then subsequently elongated using malonyl-CoA generated by ACC (Figure 3). In general, the acetyl-CoA is derived from citrate by ATP-citrate lyase (ACLY), or from acetate by acyl-coenzyme A synthetase short-chain family member 2 (ACSS2). The main product of DNL is the saturated 16 carbon fatty acid palmitate, which can be used further for the synthesis of more complex fatty acids or triglycerides as well as phospholipids by esterification with glycerol.

The interest in fructose as a driver of DNL is based on the observation that a higher dietary intake of fructose leads to an increased incidence of non-alcoholic fatty liver disease [79]. In this context, Silva and colleagues showed that, in the liver of mice, fructose contributed significantly more to saturated fatty acid synthesis compared to glucose. About 30% of fructose is used for hepatic de novo fatty acid synthesis in the form of acetyl-CoA, while the contribution of glucose is significantly lower (~10%) [80]. While this paper elegantly showed the increased fructose carbon contribution to DNL, it does not show the metabolic pathway used to convert the fructose carbon into acetyl-CoA. As shown in Figure 3, the fructose carbon backbone can enter glycolysis and can eventually be metabolized to acetyl-CoA. Recently, Zhao and colleagues, however, showed that the microbiome contributes to the supply of lipogenic acetyl-CoA by converting fructose to acetate [81]. Using in vivo stable isotope analysis, they observed that in liver-specific *Acly* knockout mice, fructose carbons were still incorporated into fatty acids and that fructose carbon contribution to hepatic acetyl-CoA was significantly reduced when the microbiome was depleted with an antibiotic cocktail. The authors concluded that dietary fructose is converted to acetate by the gut microbiome, making DNL from fructose independent of Acly activity.

Interestingly, acetyl-CoA is not the sole possibility of how fructose carbons can be utilized for lipid synthesis. Fructose carbons can end up downstream in glycerol (Figure 3), providing the backbone for triglycerides and phospholipids. Compared to glucose, it was shown that fructose provides a greater amount of carbon for G3P synthesis in liver [80]. The authors showed in ^13^C fructose fed mice that, in the liver, about 60% of newly synthesized glycerol is derived from fructose [80]. Not only is fructose preferentially metabolized to triglycerides, but it also increases triglyceride levels [82]. Triglyceride levels are elevated in several types of cancer [83,84] or associated with an increased risk of cancer [85]. These findings demonstrate that fructose is used as a carbon source for fatty acid and triglyceride synthesis and therefore provides components to sustain cancer cell growth and proliferation. However, how much oral fructose consumption actually contributes to triglyceride synthesis in healthy humans is still debated. It was estimated to contribute <1% to plasma triglycerides, but increased DNL in general as assessed by ${}^{13}$C labelled acetate studies [86], suggesting that fructose may drive DNL without donating carbons.

Over the last few years, several research groups have demonstrated that fructose consumption increases DNL [87] and expression levels of the DNL enzymes, ACC [88], FASN [89], and stearoyl CoA desaturase-1 (SCD1) [90]. In fructose-fed rats, it was observed that the expression levels of FASN and SCD1 were significantly increased compared to controls [91]. Similar effects were observed for other lipogenic enzymes [78]. Recently, it was observed that fructose, even at a moderate dose, induces fatty acid synthesis and can enhance tumorigenesis in mice [92]. All this suggests that fructose not only provides carbon for the formation of fatty acids, but also has a signaling function in DNL. However, little is known about the mechanistic role of fructose on DNL enzyme expression. A prominent regulator of DNL is sterol regulatory element-binding protein 1c (SREBF1), which induces many signals for DNL in response to carbohydrate intake [93]. In addition, carbohydrate regulatory element-binding protein (ChREBP) is activated by carbohydrate intake and controls the expression of several lipogenic enzymes [94]. In rat liver, it was observed that nucleus SREBF1 and ChREBP activity increased when dietary glucose is replaced by fructose [95]. The authors showed increased ChREBP binding to ChoRE, a promotor of lipogenic enzymes, and increased active SREBF1 [95]. It has been suggested that hexose-phosphate metabolites are important for ChREBP activation [96,97], highlighting the importance of KHK in the fructose dependent activation of ChREBP. In liver-specific *Acly* knockout mice consuming a mixture of fructose:glucose, it was observed that ChREBP and DNL gene expression regulation was independent of acetyl-CoA metabolism [81]. The results are in line with experiments showing that KHK knockout mice are protected from fructose-induced fatty liver [98,99].

A recent study shows both the carbon contribution as well as signaling effect of fructose in hepatic DNL [100]. In transgenic mice, a high-fructose diet led to higher cytosolic acetyl-CoA pool, increased liver triglycerides, and increased expression of SREBF1, ChREBP, ACC1, and FASN. The authors explained the signaling effects based on the interplay between intestinal epithelial cells, macrophages, and hepatocytes. In intestinal epithelial cells, a high-fructose diet resulted in high F1P levels and subsequently in deregulated protein *N*-glycosylation, triggering ER stress and intestinal inflammation. Inflammatory signals activated MyD88 signaling in macrophages and subsequent TNF release. TNF then induced SREBF1 signaling in hepatocytes [100].

**Figure 4:**
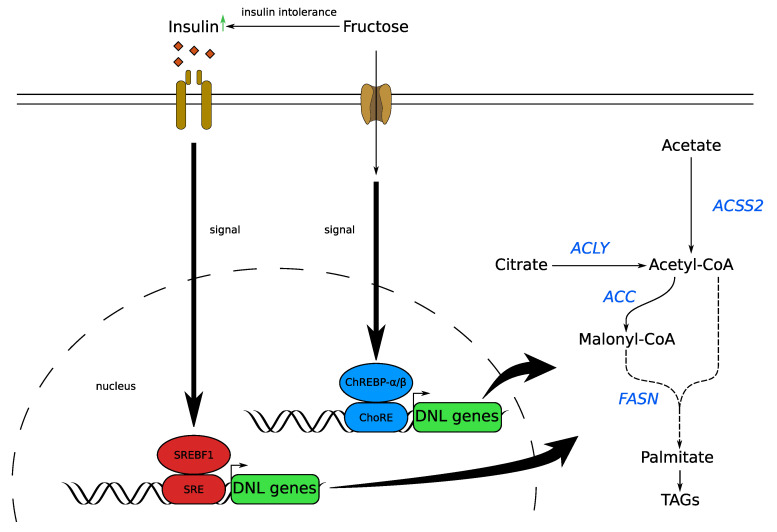
Fructose signaling effects on DNL gene expression. Fructose intake activates ChREBP, which binds to ChRE, a promotor of lipogenic emzymes. Furthermore, fructose is also involved in SREBF-mediated DNL gene expression. SREBF is activated by insulin signaling, binds to SRE, promoting gene expression of lipogenic enzmyes. Increased fructose consumption results in insulin resistance, which in turn increases insulin secretion in a compensatory manner. Therefore, fructose consumption affects DNL gene expression by ChREBP and SREBF signaling. ACC: acetyl-CoA carboxylase; ACSS2: acyl-coenzyme A synthetase short-chain family member 2; ACLY: ATP citrate lyase; AKG: α-ketoglutarate; ChREBP: carbohydrate-responsive element-binding protein; ChoRE: carbohydrate response element; DNL: de novo lipogenesis; FASN: fatty acid synthase; G3P: glycerol 3-phosphate; SRE: sterol regulatory element; SREBF: sterol regulatory element-binding protein; TCA: tricarboxylic acid cycle; TAG: triacylglyceride.

In summary, these findings suggest that, in cancer cells, fructose can play an important role as a carbon precursor for fatty acid and triglyceride synthesis as well as in the functional regulation of DNL and its gene expression (Figure 4).

In addition, DNL plays also an important role in cancer cell survival. In breast cancer cells, inhibition of ACC and FASN results in impaired cancer cell migration and invasion [101]. Furthermore, the authors showed a strong inhibition of cancer cell proliferation in breast cancer cells and hepatocellular carcinoma cells when ACC and FASN were inhibited. In addition, DNL promotes membrane lipid saturation and therefore protects cancer cells from free radicals and chemotherapeutics [102]. These findings make fructose metabolism, especially DNL, an interesting target for anti-cancer therapy and suggest that lipid synthesis play an important role in cancer cell survival and biology.

## 5. Outlook

Compared to glucose, the connection of fructose to cancer cell metabolism has long been overlooked. However, fructose can be used to generate ATP in glycolysis, donate carbon for nucleotide and lipid synthesis, and can provide signaling cues to sustain cancer cell proliferation. Indeed, in recent years, there has been mounting experimental as well as clinical evidence that increased fructose consumption can support tumorigenesis. In addition, several research groups have shown that the endogenous fructose production from glucose via the polyol pathway plays an important role in the formation of metastasis. Looking ahead, it will be interesting to quantify the metabolic flux from glucose to fructose and to subsequently follow the fate of these carbons in central metabolism. This will prove challenging, as standard metabolic flux techniques (e.g., stable-isotope assisted metabolomics) will have difficulties to distinguish the polyol pathway flux from the normal glycolytic flux. These methods need to be combined with appropriate knockout or overexpression models.

Another interesting question is whether fructose metabolism can be a suitable drug target for the treatment of cancer. Here, KHK could become an interesting therapeutic target. Many cancer cells overexpress KHK, and essential fructosuria, the genetic disorder caused by a loss of function mutation in KHK, is clinically asymptomatic and harmless, further supporting the notion that clinical inhibition of KHK may be tolerated in cancer patients.

## Abbreviations

The following abbreviations are used in this manuscript:

ACC	acetyl-CoA carboxylase
ACLY	ATP citrate lyase
ACSS2	acyl-coenzyme A synthetase short-chain family member 2
AKG	α-ketoglutarate
AKR1B1	Aldo-Keto Reductase Family 1, Member B1
ALDO	Aldolase
ChREBP	Carbohydrate-Responsive Element-Binding Protein
ChoRE	Carbohydrate-Responsive Element
DHAP	Dihydroxyacetone Phosphate
DNL	De Novo Lipogenesis
E4P	Erythrose 4-Phosphate
EMT	Epithelial-to-Mesenchymal Transition
F1P	Fructose-1-Phosphate
F6P	Fructose-6-Phosphate
FASN	Fatty Acid Synthase
F1,6BP	Fructose-1,6-Bisphosphate
FBPase	Fructose-1,6-Bisphosphatase
G3P	Glycerol-3-Phosphate
GA	Glyceraldehyde
GA3P	Glyceraldehyde-3-Phosphate
GLUT	Glucose Transporter
G6PD	Glucose-6-Phosphate Dehydrogenase
GPDH	Glycerol 3-Phosphate Dehydrogenase
GPI	Glucose-6-Phosphate Isomerase
GSH	Glutathione
GSSG	Glutathione disulfide
HFCS	High Fructose Corn Syrup
HIF1-α	Hypoxia-inducible factor 1-alpha
HK	Hexokinase
KHK	Ketohexokinase
NF-_κB_	Nuclear Factor kappa-light-chain-enhancer of activated B-cells
6PGL	6-phosphogluconolactonase
6PGDH	6-phosphogluconate dehydrogenase
PPP	Pentose Phosphate pathway
PFK	Phosphofructokinase
RPE	Ribulose 5-Phosphate 3-Epimerase
RPI	Ribose-5-Phosphate Isomerase
SCD1	Stearoyl-CoA desaturase
SORD	Sorbitol Dehydrogenase
SRE	Sterol Regulatory Element
SREBF	Sterol Regulatory Element-Binding protein
TAG	Triacylglyceride
TALDO	Transaldolase
TCA	Tricarboxylic Acid Cycle
TGFβ	Transforming Growth Factor β
TK	Triose Kinase
TKLL1	Transketolase-Like Protein
TKT	Transketolase
TNF	Tumor Necrosis Factor
TPI	Triose-Phosphate Isomerase
X5P	Xylulose 5-Phosphate
